# Genetic Etiology in Pelvic Organ Prolapse: Role of Connective Tissue Homeostasis, Hormone Metabolism, and Oxidative Stress

**DOI:** 10.3390/genes16010005

**Published:** 2024-12-24

**Authors:** Wenxuan Jiang, Rachel Yau Kar Cheung, Cheuk Yan Chung, Symphorosa Shing Chee Chan, Kwong Wai Choy

**Affiliations:** 1 Department of Obstetrics and Gynaecology, Faculty of Medicine, The Chinese University of Hong Kong, Hong Kong SAR, China; jiangwx@link.cuhk.edu.hk (W.J.); rachelcheung@cuhk.edu.hk (R.Y.K.C.); tchung19g@link.cuhk.edu.hk (C.Y.C.); symphorosa@cuhk.edu.hk (S.S.C.C.); 2 Baylor College of Medicine Joint Center for Medical Genetics, The Chinese University of Hong Kong, Hong Kong SAR, China

**Keywords:** pelvic organ prolapse, genetics, single nucleotide polymorphisms, extracellular matrix, hormone metabolism, oxidative stress

## Abstract

**Background**: Pelvic organ prolapse (POP) has become a common health problem among the aging population and affects an increasing number of elderly women worldwide. Studies within family and twin pairs provided strong evidence for the contribution of genetic factors to POP. Given the incomplete penetrance, polygenic traits, and small effect sizes of each variant in complex diseases, it is not always easy to evaluate the genetic susceptibility and molecular mechanisms involved in POP. **Objectives**: This review intends to comprehensively summarize the current studies on genetic variants associated with POP. **Methods**: We performed a comprehensive review to summarize the genetic findings from genome-linkage studies, genome-wide association studies, candidate association studies, and gene expression analyses. **Results**: We summarized genetic variants associated with connective tissue homeostasis, hormone metabolism, and oxidative stress, which were potentially related to the pathophysiology of POP. We also reviewed the limited polygenic risk score (PRS) studies generated for each individual’s genetic risk stratification and its integration into clinical risk factors for disease prediction. **Conclusions**: This pooled analysis provides moderate epidemiological credibility for associations of these genetic variants with POP to bridge the gap between genetic research and clinical medicine towards understanding the genetic etiology of POP. It also highlights the potential of PRS as a risk prediction model.

## 1. Introduction

Pelvic organ prolapse (POP) is the descent of one or more pelvic organs due to the weakness or loss of pelvic floor support from connective tissues, muscles, or both [1]. It has attracted much attention given its high prevalence, significant impairment of quality of life, and heavy emotional burden in elderly women [2,3,4]. In the United States, about 3% of women were affected by symptomatic POP [5]. In China, the prevalence of symptomatic POP was 9.6%, according to a nationwide epidemiological survey [6]. Aging in the population is a global issue that can exacerbate the burden of the economy and public healthcare systems [7]. The cumulative risk of POP surgeries was 12.6% by the age of 80 years, and the annual risk of POP surgeries increased with age until a peak of 4.3 per 1000 women between 71 and 73 years old [8]. It was predicted that, by 2050, the number of surgeries for POP will increase significantly with the aging population [9]. Therefore, understanding the pathophysiology of POP is important for us to identify women at increased risk and provide prevention and intervention strategies. Age-related biological deterioration can occur in different types of tissues and has been recognized as the main risk factor for human diseases, which results from the accumulation of cellular and molecular damage [10].

Human aging is a complex process affected by genetic and environmental factors [11]. There is a normal decline in pelvic floor function with aging. During this period, the risk factors related to the weakening or injury of pelvic floor connective tissues or muscles will accelerate the deterioration of function. The well-known factors include pregnancy, vaginal childbirth, frequency of childbirth, advanced age, hormone changes, ethnicity, increased intra-abdominal pressure, smoking, family history, and previous pelvic surgery [12]. These factors usually contribute to POP by interacting with each other rather than alone. To better understand the role of these risk factors, a “lifespan model” [13] integrated the risk factors involved physiology, anatomy, genetics, and lifestyles into three major human life phases to describe how these factors contribute to POP at different life phases and what independent, interactive, and cumulative effects they have on pelvic floor function. During an individual’s growth and development of pelvic floor function, predisposing factors could make an individual susceptible to the disease. An individual with good functional reserve may not develop POP during her lifespan, while an individual with less functional reserve may suffer from POP late or early in life based on age-related normal decline. During pregnancy and childbirth, under different predisposing conditions or interventions, some small pelvic floor injuries may completely recover, while some significant or severe pelvic floor injuries can only partially recover or cannot recover. Genetic factors can act as intrinsic drivers that regulate the growth of pelvic floor and respond to the dramatic and dynamic changes. During the aging phase, genetically programmed variations in aging, hormonal decline, and lifestyle (such as smoking and chronic cough) can cause variations in the rate of the pelvic floor function decline. Therefore, it is highlighted that genetic and environmental factors should be considered when analyzing POP causation.

In this review, we focus on the genetic factors involved in the development of POP. Recently, the International Urogynecology Consultation (IUC) evaluated the impact of genetics on POP across different studies and suggested that genetic factors had a strong etiologic link to POP [14]. Current studies are devoted to identifying the predisposed genetic variants associated with POP. Given the incomplete penetrance, polygenic traits, and small effect sizes of each variant in complex diseases, the evaluation of genetic susceptibility and molecular mechanisms involved in POP is not easy [15]. Therefore, this review intends to comprehensively summarize the current studies on genetic variants associated with POP combining with the findings from genome linkage studies, genome-wide association studies (GWASs), and candidate gene association studies in three different aspects: genetic variants associated with connective tissue homeostasis, hormone metabolism, and oxidative stress (OS).

## 2. A Familial or Genetic Basis of POP

Current epidemiological evidence demonstrates the heritability of POP, and the results are consistent between several studies. Family studies showed that prolapse could be inherited in an autosomal dominant fashion with incomplete penetrance [16]. There was also a high concordance (74.3% to 91.1%) in POP stage between nulliparous women and their parous sisters [17]. Two large, population-based studies found the relative risk (RR) of POP increased in first- and third-degree female relatives (4.15 and 1.24, respectively) and elevated from 2.36 to 6.26 with the increased number of affected first-degree relatives [18,19]. A large twin study showed that genetic, non-shared environmental, and shared environmental factors contributed to around 40%, 40%, and 20% of the variance of POP, respectively [20]. Several systematic reviews and meta-analyses showed that a positive family history of POP was associated with a 2.3- to 2.7-fold increased risk for POP as well as a 1.4- to 1.8-fold increased risk for POP recurrence [21,22,23]. These studies revealed a familial or genetic basis for POP.

## 3. Genetic Variants Associated with Remodeling of Extracellular Matrix (ECM) in Pelvic Floor Connective Tissues

ECM is a series of macromolecules composed mainly of proteins and polysaccharides, including collagens, elastin, non-collagenous glycoproteins, and proteoglycans/glycosaminoglycans [24]. ECM proteins form complex matrix structures (lamina, collagen, and elastin fibers) through direct interactions or lysyl oxidase-mediated cross-linking to maintain the strength, which is essential for maintaining homeostasis [25,26]. In addition, the degradation of extracellular proteases or the cleavage of matrix proteins can contribute to ECM degradation and remodeling [27,28]. The balance of anabolism and catabolism can keep the homeostasis of pelvic floor connective tissues and provide support for pelvic organs. The significant findings are listed in Table 1.

***Collagen*** Collagen is the main component (70–80%) of connective tissues [45]. Type I collagen and type III collagen are the main components. Type I collagen is responsible for mechanical strength, while type III collagen is responsible for tissue elasticity and extensibility [46]. Type IV collagen is the network-forming collagen that constitutes the sheet-like structure with other components [47]. Type XVIII collagen plays a role in the structure composition of the basement membranes to strengthen its structure and provide additional anchor sites [48]. Only rs1800255 of *COL3A1* was significantly associated with POP in two independent cohorts, and it was also supported by a system review and meta-analysis [29,30,49,50]. The substitution of an adenine (*A*) to guanidine (*G*) in position 2092 (exon 30) causes a missense functional variant, which leads to the disturbance of triple helical conformation of type III collagen [30]. The rs2236479 of *COL18A1* was firstly reported in a genome-wide association study by Allen-Brady et al. [44]. However, this association was not replicated in another two independent cohorts including Brazilian, African American, and Hispanic [51,52] (Supplementary Table S1). Several single nucleotide polymorphisms (SNPs) of *COL4A2*, *COL5A1*, and *COL14A1* were only identified in one small East Asian cohort [31].

***Elastic fibers and lysyl oxidase*** Elastic fiber is another important component of the pelvic connective tissues, which is exposed to extraordinary forces and provides stretching, resilience, and cell interactivity to pelvic floor tissues. Elastin and microfibrils are the main components of elastic fibers, and elastin accounts for up to 90% of their mass [53]. Elastin synthesis is a complex process, which is formed by the hierarchical assembly of its monomer (tropoelastin) [54]. Tropoelastin is secreted by elastogenic cell types and assembles into consistent globules of several microns in diameter. After excretion into the extracellular space, it undergoes rapid ordered assembly and cross-linking into elastin and is deposited on the microfibril surface [55].

Fibulins play a role in their assembly and microfibrillar deposition [54], especially fibulin-5, without which assembly would not be possible. The fibulin-5 protein is encoded by the *fibulin 5* (*FBLN5*) gene. The associations of rs12589592 and rs2018736 of *FBLN5* with POP were validated in a Russian cohort and a Chinese minority population (Table 1 and Supplementary Table S1) [32,33]. Animal studies demonstrated that *Fbln5* knockout mice had a dilated vaginal wall, enlarged genital hiatus, prolapse at more than 6 months of age, and increased severity of prolapse within 1 week postpartum [56]. In addition, fibulin-3, another component of fibulins, is encoded by the *fibulin 3* gene (*EFEMP1* gene, also known as *FBLN3*). Two genome-wide association studies also identified three SNPs related to the *EFEMP1* gene, namely, rs1430191, rs11899888, and rs3791675 [41,42]. However, these associations were not replicated in other European and Japanese cohorts [43,57].

Lysyl oxidase (LOX) is also involved in this assembly process as a catalyst [58]. The abnormal synthesis of LOX or LOX-like (LOXL) proteins leads to an altered elastogenesis. LOXL proteins 1–4 are encoded by different clusters of *LOXL* genes. Two variants, rs4886778 of *LOXL1* from a candidate gene association study and rs2862296 of *LOXL4* from a genome-wide association study, showed associations with POP [34,42]. Moreover, *LOXL4* gene, located on chromosome 10q24.2, was one of the candidate genes in the chromosome 10q24-26 region. This region was identified in a genetic linkage analysis (heterogeneity logarithm of odds [HLOD] score = 3.40) [59]. HLOD is a statistical estimate of the probability that two nearby loci are likely to be inherited together. An HLOD score higher than 3.0 is generally accepted as evidence for genetic linkage [60]. An animal study also supported that *Loxl1*-deficient mice underwent pelvic prolapse within 1–2 days postpartum and prominent pelvic descent remained over time [61].

***Laminin*** Laminin is a type of glycoproteins that forms the major non-collagenous portion of the basement membranes. Its isoforms are composed of three nonidentical chains: α, β, and γ [62]. Most isoforms contain the laminin subunit γ-1, which is encoded by *LAMC1* gene. The first genome-wide linkage analysis reported that rs10911193, located at the promoter of *LAMC1*, was associated with early-onset prolapse, potentially by affecting the binding site of the transcription factor NFIL3 (nuclear factor, interleukin 3 regulated) [63]. However, further analysis could not identify this association in different populations [33,35,64,65]. In addition, rs10911241 of *LAMC1* was shown to associate with POP in a Chinese cohort [35].

***Extracellular proteases*** Many proteases exist in the extracellular space and directly interact with the ECM, causing ECM degradation and remodeling. Degradation not only decreases the quantity of matrix proteins but also produces matrix protein degradation-derived bioactive fragments [27]. The main proteases that mediate ECM degradation include MMPs (matrix metalloproteinases), ADAMs (disintegrin and metalloproteinases), and ADAMTSs (disintegrin and metalloproteinases with thrombospondin motifs). Collagen, elastin, laminins, and proteoglycans can be degraded by these proteases as substrates. Another type of proteases, TIMPs (tissue inhibitors of matrix metalloproteinases), can inhibit the activities of MMPs, ADAMs, and ADAMTSs [66]. Several SNPs of *MMP1*, *MMP9*, and *MMP10* were associated with POP in candidate gene association studies [36,37,38,39]. Genome-wide association studies also identified the associations of rs42400 of *ADAMTS16*, rs10810888 of *ADAMTSL1*, rs235929 near *ADAMTS5*, and *ADAMTS1* with POP [42], which overlapped with hernia-associated genetic loci [67]. Other SNPs of *ADAMTS1*, *ADAMTS13*, and *TIMP2* were only found to have associations with POP in a small Chinese cohort [40]. In addition, the *TIMP2* gene, located on chromosome 17q25.3, was one of the candidate genes in the chromosome 17q25 region. This region was identified in a genetic linkage analysis (HLOD score = 3.30) [59].

In addition to the above genetic variants, some novel variants possibly related to ECM homeostasis were also identified in genome-wide association studies [42]. The rs9306894 was located at the 3’UTR region of *GDF7* gene (also known as *BMP12* gene). This gene might play a role in the differentiation of the tendon cells and promote the synthesis of type I and III collagen, which was associated with connective tissue homeostasis [68,69]. The rs11031796 of *WT1-AS* was also reported in hernia, which shared a similar pathogenesis with POP [70]. The other two loci related to the *WT1* gene were also reported in European and East Asian cohorts [43]. *HOXD13* around rs77648136 and *HOXA11* were reported to be involved in the regulation of collagen by regulating the activity of MMPs [71,72]. These findings highlighted a genetic correlation with abnormal ECM remodeling, and this progressive remodeling would contribute to the POP.

## 4. Genetic Variants Associated with Hormone Metabolism

The decline in sex hormone levels with menopause in aging women has negative effects on the female urogenital system, leading to atrophic changes. The strength and flexibility of pelvic connective tissues and muscles can also be affected by sex hormones that interact with specific receptors [73]. The sex hormone receptor is a type of nuclear hormone receptor. It is involved in transcription modulation through the ligand-binding domain (LBD) and DNA-binding domain (DBD). These two highly conserved regions can transmit activated steroid receptors from the cytoplasm to the nucleus and act on transcription regulation [74]. Except for hormone receptors, other factors related to sex hormone metabolism can also have effects on pelvic floor connective tissues. The significant findings are shown in Table 2.

Estrogen receptors include estrogen receptor α (ER-α) and estrogen receptor β (ER-β), which are encoded by the *ESR1* gene and *ESR2* gene. Estrogen regulates the transcriptional signature of its target tissues via the nuclear pathway activated by binding to ER-α and ER-β [77]. The abundance of estrogen receptors in the urogenital tract may explain why the natural reduction in endogenous estrogen can cause or potentiate pelvic floor disorders (PFDs) [78]. Only rs17847075 (rs2077647), rs2234693, and rs2228480 of *ESR1* were found to be associated with POP in Chinese and Ashkenazi-Jewish origin populations [33,65,75]. Among them, the association of rs2228480 with POP was also supported by a systematic review and meta-analysis [49]. There was no association identified between *ESR2* and POP (Supplementary Table S1). Only two haplotypes of *ESR2* were associated with POP [79]. For other types of hormone receptors, only the rs484389 located in the 3’-UTR region of progesterone receptor (*PGR*) was found to be significantly associated with POP in Chinese Taiwanese women [76].

In addition, rs3820282 of *WNT4* was identified to be associated with POP in genome-wide association studies [41,42]. This variant was also reported to increase the risk of some estrogen-related diseases [80,81]. *WNT4* is a transcription factor involved in the Wnt signaling pathway. It encodes the protein that participates in the regulation of female sexual differentiation and the development of the female reproductive tract [82]. The loss of function of *WNT4* genes leads to a partial sex reversal in humans [83]. *DVL2* (rs72839768 mapped gene) was also reported to be involved in the Wnt signaling pathway [84,85].

## 5. Genetic Variants Associated with Oxidative Stress That Disturbs Cellular Homeostasis in Pelvic Floor Support Tissues

Oxidative stress (OS) is caused by an imbalance in reactive oxygen species (ROS) and antioxidant defense systems in cells, tissues, or organs [86]. The source of ROS is mainly from the mitochondrial respiratory chain [87]. If their balance is destroyed, ROS can react spontaneously on biomolecules, such as DNA, RNA, protein, and lipids, leading to cell death and disease. Therefore, OS interferes with the process of collagen and elastin synthesis [88] (Table 3).

As shown in Table 3, two polymorphisms of OS-related genes, rs1695 of glutathione S-transferase pi 1 (*GSTP1*) and rs1136410 of poly (ADP-ribose) polymerase 1 (*PARP1*), showed significant associations with POP in the Korean population [89,90]. Glutathione S-transferases participate in the major detoxification mechanisms in humans by combining with a wide range of electrophilic compounds. *PARP1* is involved in the base excision repair of oxidative DNA damage and acts as a mediator to direct cell fates, according to the type and strength of stress [89,91]. Another three associated variants, rs1810636 near *IDH3B*, rs1036819 of *ZFAT*, and rs2267372 of *MAFF*, were also identified in genome-wide association studies [42,44]. The protein encoded by *IDH3B* is the β subunit of one isozyme of NAD(+)-dependent isocitrate dehydrogenase. It can participate in energy production by mitochondria [85]. *ZFAT* encodes a protein that likely binds to DNA and functions as a transcriptional regulator involved in apoptosis and cell survival [85]. *MAFF* encodes a basic leucine zipper (bZIP) transcription factor and acts as the OS reactive protein in the inflammation process [85,92].

## 6. Other Genetic Findings Related to Predisposition of POP

**Figure 1:**
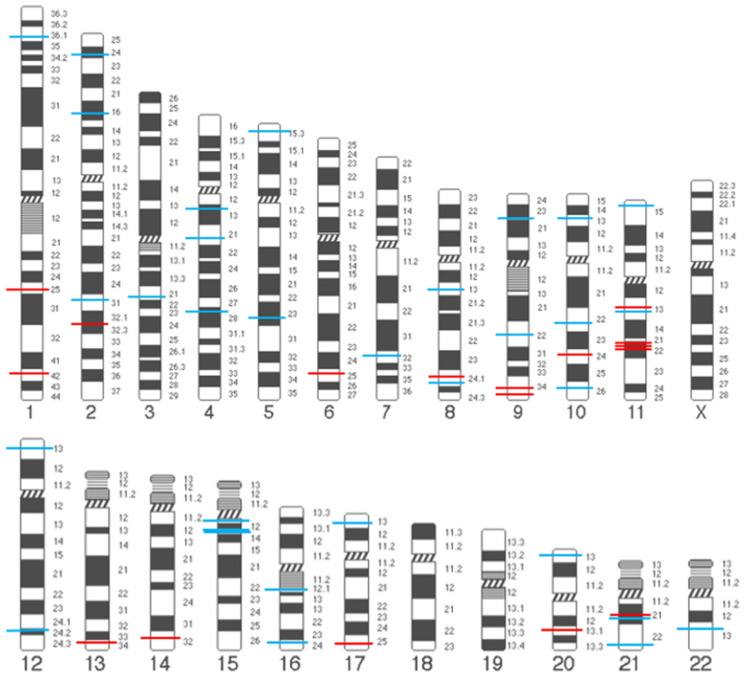
Chromosome map of significant SNPs associated with POP. Red line: the significant SNPs identified in candidate gene association study. Blue line: the significant SNPs identified in genome-wide association study.

Genome-wide association studies showed a positive correlation between metabolic and cardiovascular health and POP (rs4779517 of *KLF13*, rs12314243 of *DUSP16*, rs10762631 of *VCL*, rs7072877 near *FGFR2*, and rs73197353 and rs1247943 near *TBX5*) (Table 3) [41,42,43,92,93,94]. It indicated that the comorbidities could be risk factors of POP [95,96]. Several significant loci were identified, but the potential biological functions had not been demonstrated (Table 3). Other non-significant associations in different cohorts are listed in Supplementary Table S1 [29,30,31,32,33,34,35,36,37,38,39,40,51,63,64,65,75,76,79,88,97,98,99,100,101,102,103,104,105,106,107,108,109]. The distribution of all the significant genetic variants in chromosomes is summarized in Figure 1.

## 7. Gene Expression of POP-Associated Genes

Gene expression is an intermediate linkage between the DNA sequence and phenotype by RNA transcription and protein translation [110]. The human genome regulates these expression processes in target tissues, which varies across cell types and individuals [111]. Pelvic floor support structures mainly include ligaments, especially cardinal ligaments (CLs) and uterosacral ligaments (USLs), vaginal wall, levator ani muscle (LAM), and pelvic fasciae [112]. The increased or decreased gene expressions in these tissues may be related to the changes in pelvic floor functions. The expression of the above POP-associated genes in these pelvic floor tissues is shown in Table 4.

**Figure 2:**
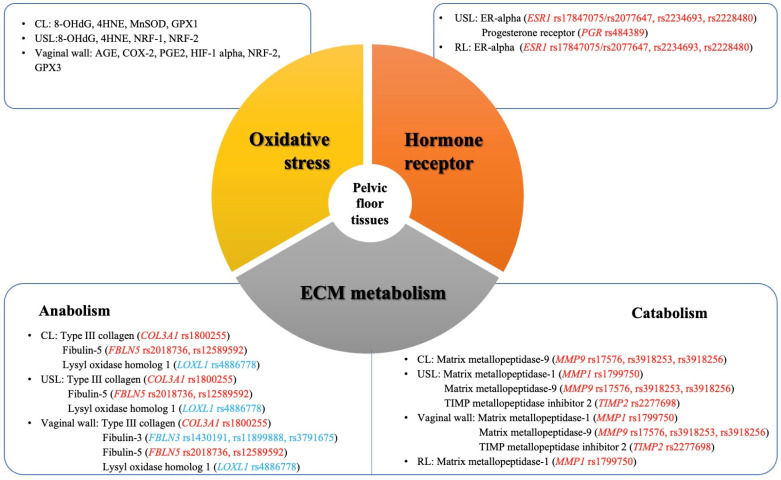
Overview of POP-associated SNPs and their related gene expression in different pelvic floor tissues. Red: significant SNPs identified in candidate gene association studies. Blue: significant SNPs identified in genome-wide association studies. Abbreviations: CL: cardinal ligament; USL: uterosacral ligament; RL: round ligament; 8-OHdG: 8-hydroxy-2’ -deoxyguanosine; 4HNE: 4-Hydroxynonenal; MnSOD: manganese superoxide dismutase; GPX1: glutathione peroxidase 1; NRF-1: nuclear respiratory factor-1; NRF-2: nuclear respiratory factor-2; AGE: advanced glycation end product; COX-2: cyclooxygenase-2; PGE2: prostaglandin E2; HIF-1 α: hypoxia-inducible factors α; GPX3: glutathione peroxidase 3.

Corresponding to the above genetic findings, for ECM-related components, there were different results regarding the RNA expression of *COL3A1* gene and the protein expression of type III collagen across different studies. The increased or decreased expressions were found in CLs, USLs, and the vaginal wall of POP subjects in different studies. However, there was no difference in round ligaments (RLs) and para-urethral tissues between POP subjects and controls [26,45,113,114,115,116,117,118,119,120,121,122,124,125,126,127,148]. The protein expression of elastin was decreased in CLs, USLs, and the vaginal wall of POP subjects in some studies [26,58,120,121,123,128,129]. The RNA expression of *FBLN5* gene and the protein expression of fibulin-5 were significantly decreased in the different tissues of POP subjects [58,113,118,130,132,133,134,135,136]. In addition, there were no differences in the RNA expression of *EFEMP1* and *LOXL4* gene and protein expression on type IV collagen, type V collagen, fibulin-3, and laminin across different tissues between POP subjects and controls [58,119,120,130,131,132,134,136]. For extracellular proteases, most studies showed an increased RNA and protein expression of *MMP1*, *MMP9*, and *MMP10* and a decreased RNA expression of *TIMP2* across different tissues in POP subjects [113,116,119,121,125,137,138,139,140,141,142,143,144,145]. For hormone receptors, the RNA expression of *ESR1* and protein expression of ER-α decreased in the USLs and RLs of POP cases [115,116,145]. However, one study found both of them increased in USLs in POP subjects [147]. The RNA and protein expressions of PGR were inconsistent in USLs [146,147]. The expressions of other genes and products of OS are shown in Supplementary Table S2 [26,45,113,114,115,116,117,118,119,120,121,122,123,124,125,126,127,128,136,137,138,141,142,143,144,145,147,148,149,150,151,152,153,154,155,156,157,158,159,160]. There was also an increased level of products of OS across different tissues in POP subjects. The significant SNPs and related gene expression in different pelvic floor tissues are shown in Figure 2. These results indicated the heterogeneity of gene expression in different tissues and cells across different populations.

Single-cell RNA sequencing (scRNA-seq) enables a deep understanding of cellular heterogeneity to some extent by analyzing all cell populations, cell-to-cell communication, and averaged gene expression at the single-cell level [161]. There were only limited studies on POP. The three most abundant cell types in the vaginal wall of POP women identified in current scRNA-seq studies were fibroblast (55.49%), smooth muscle cells (SMCs) (17.97%), and macrophages (7.51%). The genes or transcription factors in these cell types were involved in dysregulated ECM organization and immune reaction. The increased immune regulation and tissue remodeling-related interactions between these three cell types suggested that the phenotypic switch from SMCs to myofibroblasts could be the underlying cause of structural changes in the muscularis of the vaginal wall [162]. During this process, ECM organization and antigen presentation were also enhanced [163]. The age-related difference in the biological process in the vaginal wall revealed that the upregulated biological process in old POP women was mainly related to chronic inflammation, while the upregulated biological process in young POP women was mainly related to ECM metabolism [164]. Another scRNA-seq study identified that the three most abundant cell types of USLs in POP women were SMCs (34.93%), endothelial cells (21.29%), and fibroblasts (17.36%) [165]. A significant reduction in receptor–ligand pairs between immune cells and fibroblasts and cell adhesion between fibroblasts and endothelial cells were also demonstrated in the USLs of POP women.

## 8. Polygenic Risk Score (PRS) and Interaction

Although a large number of genetic variants (mostly SNPs) were identified in different association studies of POP, each variant typically has a small effect and low predictive value. A polygenic risk score (PRS) can aggregate the effects of all variants across the genome and quantify the individual’s genetic risk of developing a certain disease [15]. It is calculated by the sum of multiple risk alleles, each weighted by the corresponding effect size derived from GWAS [166]. Based on the findings from this largest genome-wide association study, the first PRS of POP was generated for each individual’s genetic risk stratification. The PRS model showed a similar prediction value with clinical joint models (five established clinical factors combined: number of children, body mass index, ever smoked, constipation, and asthma) (concordance index [Harrell C-statistic]: 0.583 ± 0.007 vs. 0.588 ± 0.007). The poor concordance index of PRS might be due to the missing heritability, such as undiscovered SNP heritability, rare variants or structural variants that are not included in the PRS, gene–gene interactions, age-dependent effects, phenotype definition, and heterogeneity of the condition, while the lower concordance index of clinical joint models might be limited by the unavailability of other clinical risk factors that would improve the predictive ability in the database, such as newborn information and mode of delivery. In addition, adding PRS to the clinical joint model could improve the prediction of the combined model (Harrell C-statistic: 0.630 ± 0.007, +4.2 percentage points) [42]. This finding illustrated PRS as a tool to evaluate individual risk prediction, but further studies need to consider adding more available factors into PRS and the clinical joint model to advance the concept of using genomic information to stratify disease risk in gynecological conditions.

## 9. Challenges and Prospects

Although current studies have identified many genetic variants associated with POP, and some related genes are expressed in pelvic floor tissues, the discordant results in different studies are still the major questions to evaluate its genetic risk. For candidate gene association studies, the inconsistencies may be explained by the small sample size and different ethnic populations. For genome-wide association studies, the “missing heritability” may be explained by common variants with small effects, rare variants with large effect sizes that are not included in the analyses, and a combination of genotypic, environmental, and epigenetic interactions [167,168]. Moreover, the identified SNPs only account for a small fraction of the genetic component, which cannot fully explain the picture of genetic characteristics [169]. Copy number variants (CNVs) are deletions or duplications of genomic fragments of more than 50 base pairs. They cover around 4.8–9.5% of the human genome and encompass more nucleotide variations than SNPs [170]. Therefore, it is proposed that CNVs can at least explain part of the “missing heritability”. Although genome-wide CNV association analyses have been applied in certain common complex diseases and successfully identified CNVs related to the risk of diseases, they are relatively limited in obstetric and gynecological diseases, especially in pelvic floor disorders. In the future, CNV-associated studies can be performed in different populations.

Current studies cannot fully correlate the genetic findings with the corresponding gene expression in pelvic floor tissues. Further studies of genetic variants in different tissues are still needed to validate these findings. Apart from the direct alteration of the DNA sequence, gene expression can also be affected by the heritable modification without any change in the DNA sequence, which is called epigenetics, including DNA methylation, histone regulation, and non-coding RNA [171]. Although current epigenetic studies on POP have identified the association of DNA methylation patterns and microRNA with POP [172,173,174], the impact of these modifiers in the development of POP and the components of pelvic floor tissues has not been demonstrated. It is necessary to consider these effects on gene expression in future studies. In addition, further studies on the relationship between genetic factors involved in connective tissues, hormone metabolism, oxidative stress and environmental factors can be performed for better understanding the contributions of genetic and environmental factors to POP.

## 10. Conclusions

Based on the current evidence, it is believed that genetic factors have a strong etiologic linkage to POP. Single nucleotide polymorphisms related to extracellular matrix homeostasis, hormone metabolism, and oxidative stress are associated with the risk of POP. The implementation of combined models with genetic and clinical risk factors can be used for each individual’s POP risk stratification. However, validation in a larger cohort is needed. Gene expression studies and epigenetic studies are also necessary for understanding their roles in the maintenance of normal functions across different tissues. Further genome-wide association studies involving copy number variants should be performed to resolve the “missing heritability” in POP.

## Figures and Tables

**Table 1:** Summary of significant single nucleotide polymorphisms (SNPs) associated with the remodeling of ECM in candidate gene association studies and genome-wide association studies.

**Candidate Gene Association Studies**											
**Component**	**SNP ID**	**Annotation**	**Change in Nucleotide**	**Candidate Gene**	**Effect Direction**		Significant Association with POP *		**Race/Ethnicity**	**Sample Size (POP/Control)**	**Reference**
Collagen	rs1800255	Exonic	*G>A*	*COL3A1*	Risk		genotype *AA*, OR 5.05		East Asian (Chinese)	84 vs. 147	[29]
					Risk		genotype *AA*, OR 5.00		European (Dutch)	202 vs. 102	[30]
	rs445348	Exonic	*A>G*	*COL4A2*	Risk		allele *G*, OR 2.15		East Asian (Chinese)	48 vs. 48	[31]
	rs76425569	Exonic	*G>A*	*COL4A2*	Risk		allele *A*, OR 2.02		East Asian (Chinese)	48 vs. 48	[31]
	rs388222	Intronic	*C>T*	*COL4A2*	Protective		allele *T*, OR 0.50		East Asian (Chinese)	48 vs. 48	[31]
	rs2281968	Intronic	*G>A*	*COL4A2*	Risk		allele *A*, OR 2.02		East Asian (Chinese)	48 vs. 48	[31]
	rs3827852	Intronic	*A>G*	*COL5A1*	Protective		allele *G*, OR 0.40		East Asian (Chinese)	48 vs. 48	[31]
	rs4870723	Exonic	*A>C*	*COL14A1*	Protective		allele *C*, OR 0.46		East Asian (Chinese)	48 vs. 48	[31]
	rs2305600	Exonic	*T>C*	*COL14A1*	Protective		allele *C*, OR 0.48		East Asian (Chinese)	48 vs. 48	[31]
	rs2305598	Exonic	*T>C*	*COL14A1*	Protective		allele *C*, OR 0.50		East Asian (Chinese)	48 vs. 48	[31]
Elastic	rs2018736	Intronic	*C>A*	*FBLN5*	Protective		allele *A*, OR 0.73		Russian	210 vs. 292	[32]
fibers	rs12589592	Intronic	*G>A*	*FBLN5*	Protective		allele *A*, OR 0.42		Russian	210 vs. 292	[32]
					Protective		genotype *AA*, OR 0.11, allele *A*, OR 0.48		East Asian (minority/non-minority Chinese)	88 vs. 108	[33]
Lysyl oxidase	rs2862296	Intergenic	*A>G*	*LOXL4*	Risk		genotype *AG*, OR 3.80; genotype *GG*, OR 4.50		East Asian (Japanese)	52 vs. 28	[34]
Laminin	rs10911241	Intronic	*A>G*	*LAMC1*	Risk		allele *G*, OR 1.71		East Asian (Chinese)	161 vs. 235	[35]
Proteases	-	Upstream Variant	*G→GG*	*MMP1*	Risk		genotype *GG/GG* (OR not analyzed)		European (Italian)	137 vs. 96	[36]
	rs17576	Exonic	*A>G*	*MMP9*	Risk		genotype *AG*, OR 5.41; genotype *GG*, OR 5.77		East Asian (Chinese)	92 vs. 152	[37]
	rs3918253	Intronic	*C>T*	*MMP9*	Risk		allele *T*, OR 1.56		Non-Hispanic White	239 vs. 197	[38]
	rs3918256	Intronic	*G>A*	*MMP9*	Risk		allele *A*, OR 1.56		Non-Hispanic White	239 vs. 197	[38]
	rs17435959	Exonic	*G>C*	*MMP10*	Risk		genotype *GC*, OR 9.59; genotype *CC*, OR 4.30		East Asian (Chinese)	91 vs. 172	[39]
	rs370850	Intronic	*C>T*	*ADAMTS1*	Risk		allele *T*, OR 3.71		East Asian (Chinese)	48 vs. 48	[40]
	rs422803	Intronic	*C>A*	*ADAMTS1*	Risk		allele *A*, OR 3.71		East Asian (Chinese)	48 vs. 48	[40]
	rs402007	5’UTR	*C>G*	*ADAMTS1*	Risk		allele *G*, OR 2.18		East Asian (Chinese)	48 vs. 48	[40]
	rs428785	Exonic	*C>G*	*ADAMTS1*	Risk		allele *G*, OR 2.18		East Asian (Chinese)	48 vs. 48	[40]
	rs434857	Exonic	*T>G*	*ADAMTS1*	Risk		allele *G*, OR 2.18		East Asian (Chinese)	48 vs. 48	[40]
	rs445784	Exonic	*G>T*	*ADAMTS1*	Risk		allele *T*, OR 2.18		East Asian (Chinese)	48 vs. 48	[40]
	rs149586801	Intronic	*C>T*	*ADAMTS13*	Protective		allele *T*, OR 0.18		East Asian (Chinese)	48 vs. 48	[40]
	rs2277698	Exonic	*C>T*	*TIMP2*	Protective		allele *T*, OR 0.37		East Asian (Chinese)	48 vs. 48	[40]
**Genome-Wide Association Study**											
**SNP ID**	**Band Region**	**Annotation**		**Effect Allele**	**Other Allele**	**EAF**	**Effect Direction**	**OR (95% CI)**	***p* Value**	**Mapped Gene(s) ^**	**Reference**
rs9306894	2p24.1	3’UTR		*G*	*A*	0.16	Risk	1.10 (1.08–1.12)	5.61 × 10^−24^	*GDF7*	[41,42]
rs1430191	2p16.1	Intergenic		*T*	*C*	0.48	Risk	1.09 (1.06–1.12)	1.00 × 10^−9^	*EFEMP1*	[41]
rs11899888	2p16.1	Intron		*G*	*A*	0.11	Risk	1.11 (1.09–1.14)	4.01 × 10^−16^	*EFEMP1*	[42]
rs3791675	2p16.1	Intron		*T*	*C*	0.25	Protective	0.92 (0.90–0.94)	1.23 × 10^−13^	*EFEMP1*	[41,42]
rs77648136	2q31.1	Intergenic		*T*	*G*	0.16	Protective	0.94 (0.92–0.96)	4.81 × 10^−8^	*HOXD13*	[42]
rs42400	5p15.32	Intergenic		*G*	*C*	0.36	Protective	0.94 (0.92–0.96)	1.65 × 10^−10^	*ADAMTS16*	[42]
rs10810888	9p22.3	Intron		*G*	*A*	0.65	Risk	1.05 (1.03–1.07)	4.00 × 10^−8^	*ADAMTSL1*	[42]
rs7072877	10q26.13	Intergenic		*C*	*T*	0.80	Risk	1.06 (1.04–1.08)	4.11 × 10^−8^	*FGFR2*	[43]
rs35166569	11p13	Intergenic		*C*	*T*	0.09	Protective	0.89 (0.86–0.93)	2.54 × 10^−8^	*WT1*	[42]
rs11031796	11p13	Intron		*A*	*G*	0.31	Protective	0.93 (0.91–0.94)	2.47 × 10^−15^	*WT1-AS*	[42]
rs10742277	11p13	Intron		*C*	*G*	0.33	Risk	1.48 (1.29–1.68)	6.72 × 10^−9^	*WT1*	[43]
rs4886778	15q24.1	Intron		*A*	*C*	0.47	Risk	1.05 (1.03–1.07)	4.12 × 10^−8^	*LOXL1*	[42]
rs235929	21q21.3	Intron		*C*	*G*	0.39	Protective	0.93 (0.92–0.95)	2.01 × 10^−12^	*ADAMTS5*, *ADAMTS1*	[42]
rs2236479	21q22.3	Intron		*A*	*G*	0.59	Risk	2.23	2.80 × 10^−7^	*COL18A1*	[44]

Abbreviations: SNP: single nucleotide polymorphism; POP: pelvic organ prolapse; OR: odds ratio; EAF: effect allele frequency; CI: confidence interval; UTR: untranslated region; * significant results were indicated by *p* < 0.05 in candidate gene association studies and by *p* < 5 × 10^−8^ in genome-wide association studies (*p* < 1 × 10^−7^ was seen as genome-wide significant in reference [44]); ^ for intergenic variants, nearby gene(s) is(are) reported.

**Table 2:** Summary of significant SNPs associated with hormone metabolism in candidate gene association studies and genome-wide association studies.

**Candidate Gene Association Studies**											
**Component**	**SNP ID**	**Annotation**	**Change in Nucleotide**	**Candidate Gene**	**Effect Direction**	Significant Association with POP *		**Race/Ethnicity**		**Sample Size (POP/Control)**	**Reference**
Estrogen receptor	rs17847075/rs2077647	Exonic	*T>C*	*ESR1*	Risk	genotype *TC*, OR 2.7		East Asian (minority/non-minority Chinese)		88 vs. 108	[33]
	rs2234693	Intronic	*T>C*	*ESR1*	Risk	genotype *TC*, OR 2.99		East Asian (minority/non-minority Chinese)		88 vs. 108	[33]
	rs2228480	Exonic	*G>A*	*ESR1*	Risk	genotype *GA*, OR 2.05		East Asian (Chinese)		88 vs. 153	[75]
					Risk	genotype *AA*, OR 39.70 genotype *GA*, OR 19.20		Ashkenazi-Jewish origin		33 vs. 33	[65]
Progestogen receptor	rs484389	Exonic	*T>C*	*PGR*	Risk	genotype *TC*, OR 4.77		East Asian (Chinese)		87 vs. 150	[76]
**Genome-Wide Association Studies**											
**SNP ID**	**Band Region**	**Annotation**		**Effect Allele**	**Other Allele**	**EAF**	**Effect Direction**	**OR (95% CI)**	***p* Value**	**Mapped Gene(s) ^**	**Reference**
rs3820282	1p36.12	Intron		*T*	*C*	0.17	Protective	0.85 (0.82–0.88)	3.30 × 10^−21^	*WNT4*	[41,42]
rs72839768	17p13.1	Exon		*A*	*G*	0.02	Risk	1.19 (1.12–1.26)	4.66 × 10^−9^	*DVL2*	[42]

Abbreviations: SNP: single nucleotide polymorphism; POP: pelvic organ prolapse; OR: odds ratio; EAF: effect allele frequency; CI: confidence interval; UTR: untranslated region; * significant results were indicated by *p* < 0.05 in candidate gene association studies and by *p* < 5 × 10^−8^ in genome-wide association studies (*p* < 1 × 10^−7^ was seen as genome-wide significant in reference [44]); ^ for intergenic variants, nearby gene(s) is(are) reported.

**Table 3:** Summary of significant SNPs associated with OS and others in candidate gene association studies and genome-wide association studies.

**Candidate Gene Association Studies**											
**Component**	**SNP ID**	**Annotation**	**Change in Nucleotide**	**Candidate Gene**	**Effect Direction**	Significant Association with POP *		**Race/Ethnicity**		**Sample Size (POP/Control)**	**Reference**
OS	rs1695	Exonic	*A>G*	*GSTP1*	Protective	genotype *AG+GG*, OR 0.63 allele *G*, OR 0.60		East Asian (Korean)		189 vs. 156	[89]
OS	rs1136410	Exonic	*T>C*	*PARP1*	Protective	genotype *CC*, OR 0.46 allele *C*, OR 0.72		East Asian (Korean)		185 vs. 155	[90]
**Genome-Wide Association Studies**											
**SNP ID**	**Band Region**	**Annotation**	**Effect Allele**	**Other Allele**		**EAF**	**Effect Direction**	**OR (95% CI)**	***p* Value**	**Mapped Gene(s) ^**	**Reference**
**OS-related**											
rs1036819	8q24.22	Intron	*C*	*A*		0.31	Risk	4.03	3.57 × 10^−21^	*ZFAT*	[44]
rs1810636	20p13	Intron	*C*	*A*		0.57	Risk	2.32	6.06 × 10^−8^	*IDH3B*	[44]
rs2267372	22q13.1	Intron	*G*	*A*		0.61	Protective	0.93 (0.91–0.95)	1.07 × 10^−13^	*MAFF*	[42]
**The variants overlapped with other metabolic and cardiovascular health**											
rs10762631	10q22.1	Intron	*A*	*G*		0.10	Protective	0.92 (0.90–0.95)	3.76 × 10^−8^	*ADK*	[42]
rs12314243	12p13.2	Intron	*T*	*C*		0.54	Protective	0.91 (0.90–0.93)	3.66 × 10^−9^	*DUSP16*	[42]
rs73197353	12q24.21	Intergenic	*C*	*T*		0.08	Risk	1.12 (1.08–1.17)	1.63 × 10^−8^	*TBX5*	[42]
rs1247943	12q24.21	Intergenic	*A*	*G*		0.12	Risk	1.09 (1.06–1.12)	1.68 × 10^−21^	*TBX5*	[41,42]
rs4779517	15q13.2	Intron	*G*	*C*		0.49	Risk	1.07 (1.05–1.09)	1.10 × 10^−11^	*KLF13*	[42]
**Others**											
rs58170120	3q21.3	Intergenic	*A*	*T*		0.18	Risk	1.08 (1.06–1.11)	1.17 × 10^−10^	*SEC61A1*	[42]
rs201194999	4q13.2	Intergenic	*T*	*C*		0.30	Protective	0.89 (0.86–0.93)	2.42 × 10^−8^	*EPHA5*	[42]
rs1455311	4q21.21	Intron	*G*	*A*		0.34	Risk	2.58	7.65 × 10^−12^	*PAQR3, BMP2K, ANTXR2*	[44]
rs28403275	4q28.1	Intergenic	*C*	*G*		0.18	Risk	1.12 (1.10–1.15)	1.58 × 10^−22^	*FAT4*	[42]
rs7682992	4q28.1	Intergenic	*T*	*A*		0.21	Risk	1.13 (1.10–1.16)	4.50 × 10^−16^	*FAT4*	[41]
rs10013769	4q28.1	Intergenic	*G*	*A*		0.65	Risk	1.07 (1.05–1.09)	1.26 × 10^−10^	*FAT4*	[42]
rs251217	5q23.3	Intron	*G*	*A*		0.61	Risk	1.06 (1.05–1.08)	4.22 × 10^−11^	*SLC12A2, FBN2*	[42]
rs72624976	7q32.1	3’UTR	*T*	*C*		0.01	Protective	0.84 (0.79–0.89)	1.14 × 10^−9^	*IMPDH1*	[41,42]
rs1493202	8q13.2	Intergenic	*G*	*T*		0.52	Risk	1.05 (1.03–1.07)	3.56 × 10^−8^	*LACTB2*	[42]
rs430794	9q22.2	Intron	*T*	*G*		0.13	Protective	0.35	6.74 × 10^−5^	*AUH*, *NFIL3*	[44]
rs6484161	11p15.4	Intron	*T*	*G*		0.31	Risk	1.06 (1.04–1.08)	5.89 × 10^−9^	*SBF2, ADM*	[42]
rs4944936	11q13.4	Intergenic	*C*	*T*		0.72	Protective	0.93 (0.91–0.95)	7.13 × 10^−12^	*CHRDL2*	[42]
rs8027714	15q11.2	Intergenic	*A*	*G*		0.26	Risk	9.04	5.65 × 10^−43^	*NPAP1*	[44]
rs12915554	15q13.1	3’UTR	*A*	*C*		0.32	Protective	0.95 (0.93–0.96)	1.06 × 10^−8^	*GREM1*	[42]
rs12325192	16q21.1	Intergenic	*T*	*C*		0.18	Protective	0.89 (0.87–0.91)	1.14 × 10^−21^	*SALL1*	[41,42]
rs1874008	16q24.1	3’UTR	*C*	*T*		0.77	Protective	0.94 (0.92–0.96)	5.77 × 10^−9^	*CRISPLD2*	[42]

Abbreviations: SNP: single nucleotide polymorphism; POP: pelvic organ prolapse; OR: odds ratio; EAF: effect allele frequency; CI: confidence interval; OS: oxidative stress; UTR: untranslated region; * significant results were indicated by *p* < 0.05 in candidate gene association studies and by *p* < 5 × 10^−8^ in genome-wide association studies (*p* < 1 × 10^−7^ was seen as genome-wide significant in reference [44]); ^ for intergenic variants, nearby gene(s) is(are) reported.

**Table 4:** Summary of POP-associated genes and their expression in different pelvic floor tissues.

Components	Tissues	Gene	RNA		Protein			Race/Ethnicity	Sample Size (POP/Control)	Reference
			^&^ Methods	^#^ POP vs. Control	Name	^&^ Methods	^#^ POP vs. Control			
**ECM-related**										
**Collagen**										
	Cardinal ligament	-	-	-	Type III collagen	IHC	↑	Caucasian	33 vs. 25	[26]
		-	-	-	Type III collagen	IHC, WB	↓	East Asian (Chinese)	30 vs. 30	[113]
	Uterosacral ligament	-	-	-	Type III collagen	IHC	↑	European (German)	25 vs. 16	[45]
		-	-	-	Type III collagen	IHC	↑	Turk	22 vs. 23	[114]
		*COL3A1*	qRT-PCR	↑	Type III collagen	IHC	↑	East Asian (Chinese)	22 vs. 34	
		*COL3A1*	qRT-PCR	ND	-	-	-	Turk	32 vs. 8	[115]
		*COL3A1*	qRT-PCR	ND	Type III collagen	IHC	ND	East Asian (Chinese)	35 vs. 20	[116]
		*COL3A1*	qRT-PCR	↓	Type III collagen	IHC	↓	East Asian (Chinese)	30 vs. 30	[117]
	Round ligament	*COL3A1*	qRT-PCR	ND	-	-	-	Turk	32 vs. 8	[115]
	Para-urethral tissues	*COL3A1*	qRT-PCR	ND	Type III collagen	IHC	ND	European (Sweden)	15 vs. 14	[118]
	Vaginal wall	-	-	-	Type III collagen	IF	↑	American	62 vs. 15	[119]
		-	-	-	Type III collagen	IHC	↓	East Asian (Chinese)	23 vs. 15	[120]
		*COL3A1*	qRT-PCR	↑	-	-	-	American	47 vs. 7	[121]
		-	-	-	Type III collagen	WB	↓	American	17 vs. 5	[122]
		-	-	-	Type III collagen	IHC	ND	Caucasian	13 vs. 13	[123]
		-	-	-	Type III collagen	IHC, IF	↑	European (Italian)	14 vs. 10	[124]
		*COL3A1*	qRT-PCR	↓	Type III collagen	IHC	↓	East Asian (Chinese)	60 vs. 35	[125]
		-	-	-	Type III collagen	IHC, WB	↑	European (Italian)	20 vs. 10	[126]
		-	-	-	Type III collagen	IHC, WB	↓	East Asian (Chinese)	35 vs. 35	[127]
		-	-	-	Type IV collagen	IHC	ND	East Asian (Chinese)	23 vs. 15	[120]
		-	-	-	Type V collagen	IF	ND	American	62 vs. 15	[119]
		-	-	-	Type V collagen	IHC	ND	East Asian (Chinese)	23 vs. 15	[120]
**Elastic fibers**										
	Cardinal ligament	-	-	-	Elastin	IHC	↓	Caucasian	33 vs. 25	[26]
	Uterosacral ligament	-	-	-	Elastin	IHC, IF	↓	European (German)	59 vs. 30	[128]
		-	-	-	Elastin	IHC	ND	East Asian (Chinese)	30 vs. 30	[58]
	Vaginal wall	-	-	-	Elastin	IHC	↓	European (Belgian)	15 vs. 0	[129]
		-	-	-	Elastin	IHC	ND	East Asian (Chinese)	23 vs. 15	[120]
		*ELN*	qRT-PCR	ND	-	-	-	American	47 vs. 7	[121]
		-	-	-	Elastin	IHC	ND	Caucasian	13 vs. 13	[123]
	Uterosacral ligament	*EFEMP1*	qRT-PCR	ND	Fibulin-3	IHC	ND	American	8 vs. 8	[130]
	Vaginal wall	*EFEMP1*	qRT-PCR	ND	Fibulin-3	IHC	ND	East Asian (Korean)	12 vs. 12	[131]
	Cardinal ligament	-	-	-	Fibulin-5	IHC	↓	East Asian (Chinese)	53 vs. 25	[132]
	Uterosacral ligament	*FBLN5*	qRT-PCR	↑	-	-	-	American	31 vs. 29	[133]
		*FBLN5*	qRT-PCR	↓	Fibulin-5	WB	↓	East Asian (Korean)	30 vs. 30	[134]
		*FBLN5*	qRT-PCR	↓	Fibulin-5	IHC	↓	American	8 vs. 8	[130]
		*FBLN5*	-	-	Fibulin-5	IHC	↓	East Asian (Chinese)	30 vs. 30	[58]
	Para-urethral tissues	*FBLN5*	qRT-PCR	↓	Fibulin-5	IHC	ND	European (Sweden)	15 vs. 14	[118]
	Vaginal wall	*FBLN5*	qRT-PCR	↓	Fibulin-5	IHC	↓	American	12 vs. 10	[135]
		*FBLN5*	qRT-PCR	ND	-	-	-	Caucasian	15 vs. 11	[136]
**Lysyl oxidase**										
	Cardinal ligament	-	-	-	Lysyl oxidase homolog 1	IHC	↓	East Asian (Chinese)	53 vs. 25	[132]
	Uterosacral ligament	*LOXL1*	qRT-PCR	↑	Lysyl oxidase homolog 1	WB	↑	East Asian (Korean)	30 vs. 30	[134]
		-	-	-	Lysyl oxidase homolog 1	IHC	↓	East Asian (Chinese)	30 vs. 30	[58]
	Vaginal wall	*LOXL1*	qRT-PCR	↓	Lysyl oxidase homolog 1	IHC, WB	ND	Caucasian	15 vs. 11	[136]
	Vaginal wall	*LOXL4*	qRT-PCR	ND	-	-	-	Caucasian	15 vs. 11	[136]
**Glycoprotein**										
	Vaginal wall	-	-	-	Laminin	IHC	ND	East Asian (Chinese)	23 vs. 15	[120]
**Extracellular proteases**										
	Uterosacral ligament	-	-	-	Interstitial collagenase	IHC	↑	European (Croatian)	40 vs. 40	[137]
		-	-	-	Interstitial collagenase	IHC	↑	European (Croatian)	46 vs. 49	[138]
		-	-	-	Interstitial collagenase	IHC	↑	Israeli	20 vs. 20	[139]
		-	-	-	Interstitial collagenase	IHC	↑	Turk	42 vs. 49	[140]
		*MMP1*	qRT-PCR	ND	Interstitial collagenase	IHC	ND	East Asian (Chinese)	35 vs. 20	[116]
	Round ligament	-	-	-	Interstitial collagenase	IHC	↑	Turk	42 vs. 49	[140]
	Vaginal wall	-	-	-	Interstitial collagenase	IHC	↑	Israeli	20 vs. 20	[139]
		*MMP1*	qRT-PCR	ND	Interstitial collagenase	IHC, IB	ND	Caucasian	17 vs. 19	[141]
		*MMP1*	qRT-PCR	↑	Interstitial collagenase	IHC	↑	East Asian (Chinese)	72 vs. 72	[142]
		*MMP1*	qRT-PCR	↑	Interstitial collagenase	IHC	↑	East Asian (Chinese)	60 vs. 35	[125]
	Cardinal ligament	-	-	-	Matrix metalloproteinase-9	IHC, WB	↑	East Asian (Chinese)	30 vs. 30	[113]
	Uterosacral ligament	-	-	-	Matrix metalloproteinase-9	IHC	↑	Israeli	20 vs. 20	[139]
		*MMP9*	qRT-PCR	↑	Matrix metalloproteinase-9	ELISA	↑	East Asian (Korean)	35 vs. 39	[143]
		-	-	-	Matrix metalloproteinase-9	IHC	ND	American	21 vs. 19	[144]
		*MMP9*	qRT-PCR	↑	Matrix metalloproteinase-9	IHC	↑	East Asian (Chinese)	35 vs. 20	[116]
	Vaginal wall	-	-	-	Matrix metalloproteinase-9	IF	↑	American	62 vs. 15	[119]
		-	-	-	Matrix metalloproteinase-9	IHC	↑	Israeli	20 vs. 20	[139]
		*MMP9*	qRT-PCR	ND	Matrix metalloproteinase-9	IHC, IB	ND	Caucasian	17 vs. 19	[141]
	Vaginal wall	*MMP10*	qRT-PCR	ND	-	-	-	American	47 vs. 7	[121]
	Uterosacral ligament	*TIMP2*	qRT-PCR	↓	Metalloproteinase inhibitor 2	IHC	↓	East Asian (Chinese)	19 vs. 9	[145]
		*TIMP2*	qRT-PCR	↓	Metalloproteinase inhibitor 2	IHC	ND	East Asian (Chinese)	35 vs. 20	[116]
	Vaginal wall	*TIMP2*	qRT-PCR	↓	Metalloproteinase inhibitor 2	IHC, IB	ND	Caucasian	17 vs. 19	[141]
	Cervix tissue	*TIMP2*	qRT-PCR	ND	Metalloproteinase inhibitor 2	IHC	ND	East Asian (Chinese)	19 vs. 9	[145]
**Hormone metabolism-related**										
**Estrogen receptor**										
	Uterosacral ligament	-	-	-	ER-α	WB	↓	East Asian (Korean)	20 vs. 24	[146]
		*ESR1*	qRT-PCR	↑	ER-α	IHC	↑	Caucasian	13 vs. 13	[147]
		*ESR1*	qRT-PCR	↓	-	-	-	Turk	32 vs. 8	[115]
		*ESR1*	qRT-PCR	↓	ER-α	IHC	↓	East Asian (Chinese)	35 vs. 20	[116]
	Round ligament	*ESR1*	qRT-PCR	↓	-	-	-	Turk	32 vs. 8	[115]
**Progesterone receptor**										
	Uterosacral ligament	-	-	-	Progesterone receptor	WB	↓	East Asian (Korean)	20 vs. 24	[146]
		*PGR*	qRT-PCR	ND	Progesterone receptor	IHC	ND	Caucasian	13 vs. 13	[147]

Abbreviations: POP: pelvic organ prolapse; ECM: extracellular matrix; ^#^ “-” indicates that it was not analyzed in original studies; “↑” indicates that there was an increase in POP group; “↓” indicates that there was an decrease in POP group; “ND” indicates that there was no difference between two groups in original studies. ^&^ Methods: IHC: immunohistochemistry; qRT-PCR: real-time reverse transcription polymerase chain reaction; WB: Western blotting; IB: immunoblotting; IF: immunofluorescence; ELISA: enzyme-linked immunosorbent assay.

## Data Availability

No new data were created or analyzed in this study. Data sharing is not applicable to this article.

## Supplementary Materials

The following supporting information can be downloaded at: https://www.mdpi.com/article/10.3390/genes16010005/s1, Table S1: Summary of non-significant genetic loci in candidate gene association studies; Table S2: Summary of gene expression of other genes in different tissues; Table S3: Gene transcript.
