# Supplementary material for: Genetic Etiology in Pelvic Organ Prolapse: Role of Connective Tissue Homeostasis, Hormone Metabolism, and Oxidative Stress
Source: Genes (Basel). 2024 Dec 24;16(1):5. doi: 10.3390/genes16010005 (PMC11765207; doi:10.3390/genes16010005)
Supplement: Supplementary file 1 [file genes-16-00005-s001.zip › genes-3340833-supplementary.pdf]

Supplementary Table S1. Summary of non-significant genetic loci in candidate gene association studies

Biological function	SNP ID	Annoatation	The change of nucleotide	Candidate gene[&]	Association with POP*	Race/ Ethnicity	Sample size (POP vs. control)	Reference
**Extracellular matrix (ECM)-related**								
Collagen	rs1800012	Intronic	*G>T*	*COL1A1*	Non-significant	White/ Non-White Brazilian	107 vs.209	[97]
					Non-significant	East Asian (Korean)	15 vs.15	[98]
					Non-significant	Caucasian/ Ashkenazi-Jewish origin	36 vs.36	[99]
					Non-significant	European (Italian)	137 vs.96	[36]
					Non-significant	White/ Black/ Mixed/ Asiatic/ Unknown	348 vs.286	[100]
					Non-significant	White/ Non-White Brazilian	112 vs.180	[101]
					Non-significant	East Asian (Japanese)	52 vs.28	[34]
	rs2586488	Intronic	*A>G*	*COL1A1*	A trend toward significance	East Asian (Chinese)	48 vs.48	[31]
	rs2249492	Intronic	*C>T*	*COL1A1*	A trend toward significance	East Asian (Chinese)	48 vs.48	[31]
	rs42524	Exonic	*C>G*	*COL1A2*	Non-significant	White/ Non-White Brazilian	112 vs.180	[102]
	rs1800255	Exonic	*G>A*	*COL3A1*	Non-significant	White/ Non-White Brazilian	111 vs.179	[103]
					Non-significant	White/ Black/ Mixed/ Asiatic/ Unknown	348 vs.286	[100]
					Non-significant	East Asian (minority/non-minority Chinese)	88 vs.108	[33]
					Non-significant	East Asian (Japanese)	52 vs.28	[34]
	rs8224	3′ UTR	*A>G*	*COL3A1*	Non-significant	European (Dutch)	202 vs.102	[30]
	rs1801183	Exonic	*C>A*	*COL3A1*	Non-significant	European (Dutch)	202 vs.102	[30]
	rs1801184	Exonic	*T>C*	*COL3A1*	Non-significant	East Asian (Chinese)	84 vs.147	[29]
	rs2236479	Intronic	*G>A*	*COL18A1*	Non-significant	White/ Black/ Mixed/ Asiatic/ Unknown	285 vs.247	[51]
	rs74941798	Exonic	*C>T*	*COL4A2*	A trend toward significance	East Asian (Chinese)	48 vs.48	[31]
	rs2305603	Exonic	*T>C*	*COL14A1*	A trend toward significance	East Asian (Chinese)	48 vs.48	[31]

	rs1050351	Exonic	*G>A*	*COL18A1*	A trend toward significance	East Asian (Chinese)	48 vs.48	[31]
	rs56335679	Intronic	*A>C*	*COL18A1*	A trend toward significance	East Asian (Chinese)	48 vs.48	[31]
	rs55690336	Intronic	*C>A*	*COL18A1*	A trend toward significance	East Asian (Chinese)	48 vs.48	[31]
Elastic fibers	rs2430339	Downstream variant	*A>G*	*FBLN5*	Non-significant	Russian	210 vs.292	[32]
	rs929608	Intronic	*T>C*	*FBLN5*	Non-significant	Russian	210 vs.292	[32]
	rs2284337	Intronic	*G>A*	*FBLN5*	Non-significant	Russian	210 vs.292	[32]
	rs2430347	Exonic	*A>G*	*FBLN5*	Non-significant	Russian	210 vs.292	[32]
	rs2430369	Intronic	*C>T*	*FBLN5*	Non-significant	Russian	210 vs.292	[32]
	rs2245701	Intronic	*A>G*	*FBLN5*	Non-significant	Russian	210 vs.292	[32]
	rs2474028	Intronic	*T>C*	*FBLN5*	Non-significant	Russian	210 vs.292	[32]
	rs2498841	Intronic	*T>G*	*FBLN5*	Non-significant	Russian	210 vs.292	[32]
	rs12586948	Intronic	*G>A*	*FBLN5*	Non-significant	Russian	210 vs.292	[32]
					Non-significant	White/ Non-White Brazilian	112 vs.180	[104]
	rs2018736	Intronic	*C>A*	*FBLN5*	Non-significant	East Asian (minority/non-minority Chinese)	88 vs.108	[33]
Lysyl oxidase	rs1048661	Exonic	*G>T*	*LOXL1*	Non-significant	White/ Non-White Brazilian	48 vs.18	[105]
	rs3825942	Exonic	*G>A*	*LOXL1*	Non-significant	White/ Non-White Brazilian	48 vs.18	[105]
	rs78803776	Exonic	*T>G*	*LOXL1*	Non-significant	White/ Non-White Brazilian	48 vs.18	[105]
	rs41429348	Exonic	*C>T*	*LOXL1*	Non-significant	White/ Non-White Brazilian	48 vs.18	[105]
	rs41435250	Exonic	*G>T*	*LOXL1*	Non-significant	White/ Non-White Brazilian	48 vs.18	[105]
	rs369758147	Exonic	*C>T*	*LOXL1*	Non-significant	White/ Non-White Brazilian	48 vs.18	[105]
	rs2165241	Intronic	*T>C*	*LOXL1*	Non-significant	East Asian (Chinese)	52 vs.28	[34]
	rs2862296	Intergenic	*A>G*	*LOXL4*	Non-significant	White/ Black/ Mixed/ Asiatic/ Unknown	285 vs.247	[51]

Laminin	rs20558	Exonic	*T>C*	*LAMC1*	Non-significant	African American/ Caucasian	165 vs.246	[63]
					A trend toward significance	Non-Hispanic White	239 vs.197	[64]
					Non-significant	East Asian (Chinese)	161 vs.235	[35]
	rs20563	Exonic	*A>G*	*LAMC1*	Non-significant	African American/ Caucasian	165 vs.246	[63]
					A trend toward significance	Non-Hispanic White	239 vs.197	[64]
					Non-significant	East Asian (minority/non-minority Chinese)	88 vs.108	[33]
					Non-significant	East Asian (Chinese)	161 vs.235	[35]
	rs10911193	Upstream Variant	*C>T*	*LAMC1*	Non-significant	African American/ Caucasian	165 vs.246	[63]
					Non-significant	Non-Hispanic White	239 vs.197	[64]
					Non-significant	Ashkenazi-Jewish origin	33 vs.33	[65]
					Non-significant	East Asian (minority/non-minority Chinese)	88 vs.108	[33]
					Non-significant	East Asian (Chinese)	161 vs.235	[35]
	rs1058177	Intronic	*A>C*	*LAMC1*	Non-significant	Non-Hispanic White	239 vs.197	[64]
	rs1413390	Intronic	*G>A*	*LAMC1*	A trend toward significance	Non-Hispanic White	239 vs.197	[64]
	rs2296291	Intronic	*C>T*	*LAMC1*	Non-significant	Non-Hispanic White	239 vs.197	[64]
	rs2483675	Intergenic	*T>A*	*LAMC1*	Non-significant	Non-Hispanic White	239 vs.197	[64]
	rs3768617	Intronic	*C>T*	*LAMC1*	Non-significant	Non-Hispanic White	239 vs.197	[64]
					Non-significant	East Asian (Chinese)	161 vs.235	[35]
	rs12041030	Intronic	*A>G*	*LAMC1*	Non-significant	Non-Hispanic White	239 vs.197	[64]
	rs12073936	Intronic	*T>G*	*LAMC1*	Non-significant	Non-Hispanic White	239 vs.197	[64]
					Non-significant	East Asian (Chinese)	161 vs.235	[35]
	rs12739316	Intronic	*G>T*	*LAMC1*	Non-significant	Non-Hispanic White	239 vs.197	[64]
	rs41475048	Intronic	*T>G*	*LAMC1*	Non-significant	Non-Hispanic White	239 vs.197	[64]
	rs10911206	Intronic	*A>C*	*LAMC1*	Non-significant	Non-Hispanic White	239 vs.197	[64]
	rs10911211	Intronic	*A>T*	*LAMC1*	Non-significant	Non-Hispanic White	239 vs.197	[64]

	rs10911214	Intronic	*T>C*	*LAMC1*	Non-significant	East Asian (Chinese)	161 vs.235	[35]
	rs729819	Intronic	*G>A*	*LAMC1*	Non-significant	East Asian (Chinese)	161 vs.235	[35]
	rs869133	Intronic	*G>C*	*LAMC1*	Non-significant	East Asian (Chinese)	161 vs.235	[35]
	rs6424889	Intronic	*G>C*	*LAMC1*	Non-significant	East Asian (Chinese)	161 vs.235	[35]
Extracellular Proteases	--	Upstream Variant	*G>GG*	*MMP1*	Non-significant	European (Pole)	133 vs.132	[106]
	rs3025058	Upstream Variant	*AAAAA>AAAAAA*	*MMP3*	Non-significant	European (Italian)	137 vs.96	[36]
					Non-significant	European (Pole)	133 vs.132	[106]
					Non-significant	European (Greek)	80 vs.80	[107]
					Non-significant	White/ Non-White Brazilian	112 vs.180	[108]
	rs17576	Exonic	*A>G*	*MMP9*	Non-significant	Non-Hispanic White	239 vs.197	[38]
					Non-significant	East Asian (minority/non-minority Chinese)	88 vs.108	[33]
	rs17577	Exonic	*G>A*	*MMP9*	Non-significant	Non-Hispanic White	239 vs.197	[38]
	rs2250889	Exonic	*G>C*	*MMP9*	Non-significant	East Asian (Chinese)	92 vs.152	[37]
	rs2236416	Intronic	*A>G*	*MMP9*	Non-significant	Non-Hispanic White	239 vs.197	[38]
	rs2274755	Intronic	*G>T*	*MMP9*	Non-significant	Non-Hispanic White	239 vs.197	[38]
	--	Upstream Variant	*C>T*	*MMP9*	Non-significant	European (Italian)	137 vs.96	[36]
					Non-significant	White/ Non-White Brazilian	86 vs.158	[109]
	rs3787268	Intronic	*G>A*	*MMP9*	Non-significant	Non-Hispanic White	239 vs.197	[38]
	rs3918242	Upstream Variant	*C>T*	*MMP9*	Non-significant	East Asian (Chinese)	92 vs.152	[37]
	rs3918254	Intronic	*C>T*	*MMP9*	Non-significant	East Asian (Chinese)	48 vs.48	[40]

	rs3918278	Upstream Variant	*G>A*	*MMP9*	Non-significant	Non-Hispanic White	239 vs.197	[38]
	rs17293607	Exonic	*C>T*	*MMP10*	Non-significant	East Asian (Chinese)	91 vs.172	[39]
	rs3758853	Intronic	*C>G*	*MMP13*	Non-significant	East Asian (Chinese)	48 vs.48	[40]
	rs78356340	Intronic	*G>A*	*MMP13*	Non-significant	East Asian (Chinese)	48 vs.48	[40]
	rs391253	Intergenic	*T>C*	*MMPS*	Non-significant	East Asian (minority/non-minority Chinese)	88 vs.108	[33]
	rs9862	Exonic	*T>C*	*TIMP3*	Non-significant	East Asian (Chinese)	48 vs.48	[40]
	rs10433537	Intronic	*A>T*	*TIMP4*	Non-significant	East Asian (Chinese)	48 vs.48	[40]
	rs436525	Exonic	*G>A*	*ADAMTS1*	Non-significant	East Asian (Chinese)	48 vs.48	[40]
	rs1055432	Exonic	*C>A*	*ADAMTS13*	Non-significant	East Asian (Chinese)	48 vs.48	[40]
	rs4747097	Intronic	*C>T*	*ADAMTS14*	Non-significant	East Asian (Chinese)	48 vs.48	[40]
	**Hormone metabolism-related**							
Estrogen receptor	rs17847075	Exonic	*T>C*	*ESR1*	Non-significant	East Asian (Chinese)	88 vs.153	[75]
	rs2234693	Intronic	*T>C*	*ESR1*	Non-significant	East Asian (Chinese)	88 vs.153	[75]
	rs3798577	Intronic	*T>C*	*ESR1*	Non-significant	East Asian (Chinese)	88 vs.153	[75]
	rs2228480	Exonic	*G>A*	*ESR1*	Non-significant	East Asian (minority/non-minority Chinese)	88 vs.108	[33]
	rs2987983	Intronic	*T>C*	*ESR2*	Non-significant	East Asian (Chinese)	69 vs.141	[79]
					Non-significant	East Asian (minority/non-minority Chinese)	88 vs.108	[33]
	rs1271572	Intronic	*G>T*	*ESR2*	Non-significant	East Asian (Chinese)	69 vs.141	[79]
					Non-significant	East Asian (minority/non-minority Chinese)	88 vs.108	[33]
	rs944459	Intronic	*C>T*	*ESR2*	Non-significant	East Asian (Chinese)	69 vs.141	[79]
	rs1256049	Exonic	*G>A*	*ESR2*	Non-significant	East Asian (Chinese)	69 vs.141	[79]
					Non-significant	East Asian (minority/non-minority Chinese)	88 vs.108	[33]

	rs1255998	Non coding transcript variant	*G>C*	*ESR2*	Non-significant	East Asian (Chinese)	69 vs.141	[79]
Progestogen receptors	rs500760	Exonic	*A>G*	*PGR*	Non-significant	East Asian (Chinese)	87 vs.150	[76]
					Non-significant	East Asian (minority/non-minority Chinese)	88 vs.108	[33]
	rs484389	Non coding transcript variant	*T>C*	*PGR*	Non-significant	East Asian (minority/non-minority Chinese)	88 vs.108	[33]

Abbreviations: SNP: single nucleotide polymorphism; POP: pelvic organ prolapse; OR: odds ratio

*Non-significant results in candidate gene association studies were indicated by *P>0.05*.

A trend toward significance was indicated by 0.05< *P* <0.01 according to the definition of original studies.

Supplementary Table S2. Summary of gene expression of other genes in different tissues

Components	Tissues	Genes	RNA		Protein			Race/ Ethnicity	Sample size (POP vs. control)	Reference
			&Methods&	#POP vs. Control	Name	&Methods	#POP vs. Control			
**ECM-related**										
**Collagen**										
	Cardinal ligament	-	-	-	Type I collagen	IHC	ND	Caucasian	33 vs.25	[26]
		-	-	-	Type I collagen	ELISA	ND	Turk	22 vs.23	[149]
		-	-	-	Type I collagen	IHC; WB	ND	East Asian (Chinese)	30 vs.30	[113]
	Uterosacral ligament	-	-	-	Type I collagen	IHC	ND	European (German)	25 vs.16	[45]
		-	-	-	Type I collagen	IHC	↓	Croatian	46 vs.49	[138]
		-	-	-	Type I collagen	IHC	↓	Turk	39 vs.35	[114]
		-	-	-	Type I collagen	ELISA	↓	Turk	22 vs.23	[149]
		COL1A1	qRT-PCR	ND	-	-	-	Turk	32 vs.8	[115]
		COL1A1	qRT-PCR	↓	Type I collagen	IHC	↓	East Asian (Chinese)	35 vs.20	[116]
		-	-	-	Type I collagen	IHC	↓	Indonesian	22 vs.22	[150]
		COL1A1	qRT-PCR	↓	Type I collagen	IHC	↓	East Asian (Chinese)	30 vs.30	[117]
	Round ligament	*COL1A1*	qRT-PCR	ND	-	-	-	Turk	32 vs.8	[115]
	Para-urethral tissues	*COL1A1*	qRT-PCR	ND	Type I collagen	IHC	ND	European (Sweden)	15 vs.14	[118]
	Vaginal wall	-	-	-	Type I collagen	IF	ND	American	62 vs.15	[119]
		-	-	-	Type I collagen	IHC	ND	East Asian (Chinese)	23 vs.15	[120]
		COL1A1	qRT-PCR	↑	-	-	-	American	47 vs.7	[121]
		-	-	-	Type I collagen	WB	ND	American	17 vs.5	[122]
		-	-	-	Type I collagen	IHC	ND	Caucasian	13 vs.13	[123]

	-	-	-	Type I collagen	IHC; WB	↓	East Asian (Chinese)	44 vs.46	[151]
	-	-	-	Type I collagen	IHC; IF	↓	European (Italian)	14 vs.10	[124]
	COL1A1	qRT-PCR	↓	Type I collagen	IHC	↓	East Asian (Chinese)	60 vs.35	[125]
	-	-	-	Type I collagen	IHC; WB	↓	European (Italian)	20 vs.10	[126]
	-	-	-	Type I collagen	IHC; WB	↓	East Asian (Chinese)	35 vs.35	[127]
	-	-	-	Type I collagen	WB	ND	American	17 vs.5	[122]
Vaginal wall	-	-	-	Type VI collagen	IHC	ND	East Asian (Chinese)	23 vs.15	[120]
**Lysyl oxidase**									
Vaginal wall	*LOXL2*	qRT-PCR	ND	Lysyl oxidase homolog 2	IHC; IB	ND	Caucasian	15 vs.11	[136]
Vaginal wall	*LOXL3*	qRT-PCR	↓	Lysyl oxidase homolog 3	IHC; IB	↓	Caucasian	15 vs.11	[136]
**Glycoprotein**									
Para-urethral tissues	*FBN1*	qRT-PCR	ND	Fibrillin-1	ND	IHC	European (Sweden)	15 vs.14	[118]
	FBN1	qRT-PCR	ND	-	-	-	Turk	30 vs.30	[152]
Vaginal wall	*FBN1*	qRT-PCR	ND	-	-	-	Caucasian	15 vs.11	[136]
Vaginal wall	*FBN2*	qRT-PCR	ND	-	-	-	Caucasian	15 vs.11	[136]
**Extracellular Proteases**									
Cardial ligament	*MMP2*	qRT-PCR	↑	-	-	-	East Asian (Chinese)	15 vs.15	[154]
Uterosacral ligament	-	-	-	72 kDa type IV collagenase	IHC	ND	European (Croatian)	40 vs.40	[137]
	MMP2	qRT-PCR	↑	72 kDa type IV collagenase	IHC	↑	East Asian (Chinese)	19 vs.9	[145]
	MMP2	qRT-PCR	↑	72 kDa type IV collagenase	ELISA	↑	East Asian (Korean)	35 vs.39	[143]
	MMP2	qRT-PCR	↑	-	-	-	Turk	18 vs.15	[155]
	MMP2	qRT-PCR	↑	72 kDa type IV collagenase	IHC	↑	East Asian (Chinese)	22 vs.34	[148]
	MMP2	qRT-PCR	↑	72 kDa type IV collagenase	IHC	↑	East Asian (Chinese)	35 vs.20	[116]
Vaginal wall	-	-	-	72 kDa type IV collagenase	IF	ND	American	62 vs.15	[119]
	MMP2	qRT-PCR	↑	72 kDa type IV collagenase	IHC; IB	↑	Caucasian	17 vs.19	[141]

	MMP2	qRT-PCR	↑	72 kDa type IV collagenase	IHC	↑	East Asian (Chinese)	72 vs.72	[142]
	-	-	-	72 kDa type IV collagenase	IHC; WB	↑	East Asian (Chinese)	35 vs.35	[127]
Uterosacral ligament	*MMP3*	qRT-PCR	↑	Stromelysin-1	IHC	↑	East Asian (Chinese)	30 vs.30	[117]
Vaginal wall	*MMP3*	qRT-PCR	ND	-	-	-	American	47 vs.7	[121]
	MMP3	qRT-PCR	↑	Stromelysin-1	IHC	↑	East Asian (Chinese)	72 vs.72	[142]
	-	-	-	Stromelysin-1	IHC	↑	European (Italian)	14 vs.10	[124]
	-	-	-	Stromelysin-1	IHC; WB	↑	European (Italian)	20 vs.10	[126]
Vaginal wall	*MMP8*	qRT-PCR	↑	Neutrophil collagenase	IHC	↑	East Asian (Chinese)	60 vs.35	[125]
Vaginal wall	*MMP11*	qRT-PCR	ND	-	-	-	American	47 vs.7	[121]
Vaginal wall	*MMP12*	qRT-PCR	ND	Macrophage metalloelastase	IHC; IB	ND	Caucasian	17 vs.19	[141]
Cardinal ligament	-	-	-	Metalloproteinase inhibitor 1	IHC; WB	↓	East Asian (Chinese)	30 vs.30	[113]
Uterosacral ligament	*TIMP1*	qRT-PCR	ND	Metalloproteinase inhibitor 1	IHC	ND	East Asian (Chinese)	19 vs.9	[145]
	TIMP1	qRT-PCR	↓	Metalloproteinase inhibitor 1	IHC	ND	East Asian (Chinese)	35 vs.20	[116]
Vaginal wall	*TIMP1*	qRT-PCR	↓	Metalloproteinase inhibitor 1	IHC	↓	Caucasian	17 vs.19	[141]
	TIMP1	qRT-PCR	↓	Metalloproteinase inhibitor 1	IHC	↓	East Asian (Chinese)	72 vs.72	[142]
	-	-	-	Metalloproteinase inhibitor 1	IHC	↓	European (Italian)	14 vs.10	[124]
	TIMP1	qRT-PCR	↓	Metalloproteinase inhibitor 1	IHC	↓	East Asian (Chinese)	60 vs.35	[125]
	-	-	-	Metalloproteinase inhibitor 1	IHC; WB	↓	East Asian (Chinese)	35 vs.35	[127]
Cardinal ligament	-	-	-	Metalloproteinase inhibitor 3	ELISA	ND	Turk	22 vs.23	[149]
Uterosacral ligament	*TIMP3*	qRT-PCR	ND	Metalloproteinase inhibitor 3	IHC	ND	East Asian (Chinese)	19 vs.9	[145]
	-	-	-	Metalloproteinase inhibitor 3	ELISA	↓	Turk	22 vs.23	[149]
Vaginal wall	*TIMP3*	qRT-PCR	↓	Metalloproteinase inhibitor 3	IHC	ND	Caucasian	17 vs.19	[141]
Vaginal wall	*TIMP3*	qRT-PCR	↓	Metalloproteinase inhibitor 4	IHC; IB	ND	Caucasian	17 vs.19	[141]

Cardinal ligament	-	-	-	A disintegrin and metalloproteinase with thrombospondin motifs 2	ELISA	ND	Turk	22 vs.23	[149]
Uterosacral ligament	-	-	-	A disintegrin and metalloproteinase with thrombospondin motifs 2	ELISA	↓	Turk	22 vs.23	[149]
Vaginal wall	*ADAMTS2*	qRT-PCR	↑	A disintegrin and metalloproteinase with thrombospondin motifs 2	IHC; IB	ND	Caucasian	17 vs.19	[141]
**Others**									
Uterosacral ligament	*HOXA11*	qRT-PCR	ND	-	-	-	Turk	18 vs.15	[155]
	HOXA11	qRT-PCR	ND	-	-	-	Turk	32 vs.8	[115]
	HOXA11	qRT-PCR	↓	-	-	-	Turk	32 vs.8	[115]
Round ligament	*HOXA11*	qRT-PCR	ND	-	-	-	Turk	32 vs.8	[115]
Uterosacral ligament	*HOXA13*	qRT-PCR	↓	-	-	-	Turk	32 vs.8	[115]
Round ligament	*HOXA13*	qRT-PCR	ND	-	-	-	Turk	32 vs.8	[115]
Vaginal wall	*HOXA13*	qRT-PCR	↓	Homeobox protein Hox-A13	IHC	↓	American	36 vs.11	[156]
**Hormone metabolism-related**									
Estrogen receptor									
Uterosacral ligament	*ESR2*	qRT-PCR	ND	ER-beta	IHC	↑	Caucasian	13 vs.13	[147]
	ESR2	qRT-PCR	ND	-	-	-	Turk	32 vs.8	[115]
	ESR2	qRT-PCR	ND	ER-beta	IHC	ND	East Asian (Chinese)	35 vs.20	[116]
Round ligament	*ESR2*	qRT-PCR	ND	-	-	-	Turk	32 vs.8	[115]
**OS-related**									
Vaginal wall	-	-	-	AGE (Advanced glycation end product)	IHC; WB	↑	European (Italian)	20 vs.10	[126]
	-	-	-	AGE (Advanced glycation end product)	IHC; WB	↑	East Asian (Chinese)	44 vs.46	[151]

Vaginal wall	-	-	-	RAGE (Advanced glycosylation end product-specific receptor)	IHC; WB	Absent	European (Italian)	20 vs.10	[126]
	-	-	-	RAGE (Advanced glycosylation end product-specific receptor)	IHC; WB	ND	East Asian (Chinese)	44 vs.46	[151]
Cardinal ligament	-	-	-	8-OHdG (8-hydroxy-2' -deoxyguanosine)	IHC	↑	East Asian (Chinese)	40 vs.20	[157]
Uterosacral ligament	-	-	-	8-OHdG (8-hydroxy-2' -deoxyguanosine)	IHC	↑	East Asian (Korean)	26 vs.29	[158]
Cardinal ligament	-	-	-	4HNE (4-Hydroxynonenal)	IHC	↑	East Asian (Chinese)	40 vs.20	[157]
Uterosacral ligament	-	-	-	4HNE (4-Hydroxynonenal)	IHC	↑	East Asian (Korean)	26 vs.29	[158]
Vaginal wall	-	-	-	COX-2 (Cyclooxygenase-2)	IHC; WB	↑	East Asian (Chinese)	35 vs.35	[127]
Vaginal wall	-	-	-	PGE2 (Prostaglandin E2)	IHC; WB	↑	East Asian (Chinese)	35 vs.35	[127]
Vaginal wall	-	-	-	HIF-1 alpha (Hypoxia-inducible factor-1 alpha)	IHC	↑	European (Croatian)	60 vs.60	[159]
Uterosacral ligament	-	-	-	NRF-1 (Nuclear respiratory factor 1)	IHC; WB	ND	East Asian (Chinese)	33 vs.38	[127]
Uterosacral ligament	-	-	-	NRF-2 (Nuclear factor erythroid 2-related factor 2)	IHC; WB	ND	East Asian (Chinese)	33 vs.38	[127]
Vaginal wall	-	-	-	NRF-2 (Nuclear factor erythroid 2-related factor 2)	IHC; WB	↓	East Asian (Chinese)	35 vs.35	[127]
Cardinal ligament	*MnSOD*	qRT-PCR	↑	Superoxide dismutase [Mn], mitochondrial	WB	↓	East Asian (Chinese)	40 vs.20	[157]
Cardinal ligament	*GPX1*	qRT-PCR	↑	Glutathione peroxidase 1	WB	↑	East Asian (Chinese)	40 vs.20	[157]
Vaginal wall	-	-	-	Glutathione peroxidase3	IHC; WB	↓	East Asian (Chinese)	35 vs.35	[127]

Abbreviations: POP: pelvic organ prolapse; ECM: extracellular matrix; OS: oxidative stress

"-" indicated that it was not analyzed in original studies; "↑" indicates that there was an increase in POP group; "↓" indicates that there was an decrease in POP group; "ND" indicated that there was no difference between two groups in original studies.

[&] Methods: IHC: immunohistochemistry; qRT-PCR: Real-Time revers e transcription polymerase chain reaction; WB: Western blotting; IB: immunoblotting; IF: Immunofluorescence

Supplementary Table S3. Gene transcripts

Candidate gene	Transcript	Candidate gene	Transcript
COL1A1	NM_000088.4	*GDF7*	NM_182828.4
COL1A2	NM_000089.4	*EFEMP1*	NM_001039348.3
COL3A1	NM_000090.4	*WT1*	NM_024426.6
COL4A2	NM_001846.4	*WT1-AS*	NR_023920.2
COL5A1	NM_000093.5	*ESR1*	NM_000125.4
COL14A1	NM_021110.4	*ESR2*	NM_001437.3
COL18A1	NM_001379500.1	*PGR*	NM_000926.4
FBLN5	NM_006329.4	*WNT4*	NM_030761.5
LOXL1	NM_005576.4	*DVL2*	NM_004422.3
LAMC1	NM_002293.4	*GSTP1*	NM_000852.4
MMP1	NM_002421.4	*PARP1*	NM_001618.4
MMP3	NM_002422.5	*ZFAT*	NM_020863.4
MMP9	NM_004994.3	*MAFF*	NM_012323.4
MMP10	NM_002425.3	*ADK*	NM_006721.4
MMP13	NM_002427.4	*DUSP16*	NM_030640.3
TIMP2	NM_003255.5	*KLF13*	NM_015995.4
TIMP3	NM_000362.5	*SLC12A2*	NM_001046.3
TIMP4	NM_003256.4	*IMPDH1*	NM_000883.4
ADAMTS1	NM_006988.5	*SBF2*	NM_030962.4
ADAMTS13	NM_139027.6	*GREM1*	NM_013372.7
ADAMTS14	NM_080722.4	*CRISPLD2*	NM_031476.4
ADAMTSL1	NM_001040272.6		
